# Profil bactériologique des sinusites maxillaires chroniques suppurées d'origine nasale de l'adulte au CHU Tokoin de Lomé

**DOI:** 10.11604/pamj.2013.16.48.1210

**Published:** 2013-10-11

**Authors:** Amana Bathokédéou, Dagnra Anoumou Yaotse, Pegbessou Essobozou, Kpemissi Eyawelhon

**Affiliations:** 1 Service ORL CHU-Tokoin, Togo; 2 Laboratoire de bactériologie CHU-Tokoin, Togo

**Keywords:** sinusite, maxillaire, épidémiologie, microbiologie, sinusitis, maxillary, epidemiology, microbiology

## Abstract

Le but de cette étude était de déterminer les germes responsables des sinusites maxillaires chroniques et leur sensibilité aux antibiotiques usuels. Il s'agit d'une étude prospective menée sur 24 mois chez des patients reçus pour sinusite maxillaire chronique suppurée d'origine nasale. L'examen bactériologique était réalisé sur des prélèvements de pus obtenus par ponction des sinus. Sur les 100 prélèvements, 64 cultures (64%) étaient positives, dont 60 cultures étaient monomicrobiennes. Les germes les plus fréquemment isolés étaient le *Streptococcus pneumoniae* dans 20 cas (27.78%), le *Staphylococcus aureus* dans 15 cas (20.83%) et les streptocoques du groupe A dans 10 cas (13.89%). Les souches de *Streptococcus pneumoniae* étaient toutes sensible aux céphalosporines, aux quinolones, aux macrolides et à l'oxacilline. Les souches de *Staphylococcus aureus* avaient présenté 93.34% de résistance à la pénicilline G. Aucune souche n’était résistante à l'Oxacilline. Les streptocoques du groupe A avaient une sensibilité de 80 à 100% aux bétalactamines et aux macrolides, et 60% aux quinolones. L’écologie bactérienne des sinusites maxillaires chroniques suppurées de l'adulte est dominée par le *Streptococcus pneumoniae* et le *Staphylococcus aureus*. Ces germes sont sensibles aux quinolones et aux aminosides.

## Introduction

Les sinusites sont des affections courantes dans les services d'Oto-rhino-laryngologie (ORL). La sinusite s'observe à tout âge, mais la sinusite maxillaire est plus fréquente chez l'adulte [1], et devient chronique lorsqu'elle dure plus de trois mois. Le polymorphisme extrême de la sinusite maxillaire lui fait prendre les aspects les plus divers, selon l’évolution (aiguë, subaiguë ou chronique), la cause (infection nasale, infection dentaire, allergie), l'allure (congestive, suppurée ou séreuse), l'extension à un ou plusieurs sinus de l'un ou des deux côtés et selon les symptômes (évidente ou latente, révélée seulement par des manifestations à distance). L'examen bactériologique des secrétions sinusiennes joue un rôle fondamental dans l'affirmation de l’étiologie bactérienne des sinusites et l'orientation thérapeutique par la réalisation des antibiogrammes. L'antibiothérapie probabiliste dans la prise en charge des sinusites maxillaires chroniques repose sur une étude préalable de l’écologie bactérienne du milieu. Cette étude était initiée afin de déterminer le profil bactériologique des sinusites maxillaires chroniques suppurées d'origine nasale de l'adulte, d’étudier la sensibilité de ces germes vis-à-vis des antibiotiques usuels, et proposer un schéma thérapeutique probabiliste en cas de sinusite aux médecins généralistes.

## Méthodes

L’étude était prospective, allant du 1^er^ juin 2007 au 31 mai 2009 (soit 2 ans). Elle portait sur 100 patients âgés de plus 15 ans, vus en consultation externe ORL et qui présentaient les signes cliniques de sinusite maxillaire évoluant depuis plus de 3 mois, dont une origine nasale était confirmée par des examens ORL et odontologique. Tous les patients avaient bénéficié d'une radiographie de la face en incidence de Blondeau qui montrait des signes de sinusite maxillaire collectée. Chaque patient avait subi une ponction du sinus maxillaire au moins deux semaines après l'arrêt de toute antibiothérapie. Le prélèvement du pus était réalisé par aspiration à l'aide d'une seringue stérile à travers le trocart de ponction et acheminé dans un délai de 10 mn au laboratoire de bactériologie. Les prélèvements étaient systématiquement ensemencés sur gélose au sang cuit + Isovitalex, gélose au sang à l'acide nalidixique, gélose à l'Eosine bleu de méthylène et bouillon thioglycolate. Les milieux étaient incubés à 37°C pendant 48 heures au minimum. L'identification des bactéries était faite sur des caractères morphologiques, culturaux et biochimiques. Les antibiogrammes étaient réalisés selon la méthode de diffusion sur gélose de Mueller-Hinton. Les résultats étaient interprétés selon les règles et les recommandations du comité d'antibiogramme de la société française de microbiologie.

## Résultats

Les sinusites maxillaires chroniques représentaient 5.01% des consultations du service d'ORL. L’âge des patients variait de 16 à 79 ans avec une moyenne d’âge de 34,08 ans. Le sex-ratio était de 0.92 (d'une femme pour un homme). La sinusite maxillaire chronique était unilatérale dans 62 cas (62%) et à localisation droite dans 32 cas (32%). Sur les 100 prélèvements 64 cultures étaient positives. Soixante cultures étaient monomicrobiennes et quatre cultures polymicrobiennes, il s'agissait de l'association *Proteus vulgaris-Streptococcus pneumoniae (S. pneumoniae)* dans un cas et *Staphylococcus aureus (S. aureus)-S. pneumoniae* dans 3 cas. *S. pneumoniae* et les *S. aureus* étaient les germes les plus fréquents (Tableau 1). Les souches de *S. pneumoniae* isolées étaient sensibles aux aminosides, et aux betalactamines.

**Tableau 1:** Répartition des 72 bactéries isolées des sinusites maxillaires

	Effectif	Pourcentage
*Streptococcus pneumoniae*	20	27.78
*Staphylococcus aureus*	15	20.83
Enterobactéries*	11	15.28
Streptocoque du groupe A	10	13.89
*Pseudomonas aeruginosa*	08	11.11
Staphylocoque à coagulase négative	08	11.11
Total	**72**	**100**

* Proteus spp = 6 cas, Citrobacter spp = 3 cas, Escherichia coli = 2 cas.

Toutes les souches de S. aureus étaient sensibles à l'oxacilline et à l'association amoxicilline-acide clavulanique. Treize souches étaient sensibles aux quinolones et aux aminosides (Tableau 2). Les Streptocoques du groupe A avaient une bonne sensibilité aux betalactamines (Tableau 2) et les entérobacteries isolées étaient sensibles aux céphalosporines de 3^e^ génération, aux animosides et aux quinolones et résistantes aux autres bètalactamines (ampicilline, association amoxicilline- acide clavulanique, cépholosporine de 1^ère^ et 2^e^ génération).

**Tableau 2:** Sensibilité aux antibiotiques des Pneumocoques, des Staphylococcus aureus et des Streptocoques

		*S. pneumoniae* N = 20	*S. aureus* N = 15	Streptocoque du groupe A N = 10
**Betalactamines**	Penicilline G	12 (60%)	1 (6.66%)	10 (100%)
	Oxacilline (100%)	20	15 (100%)	8 (80%)
	Amoxicilline + Ac cl	20 (100%)	15 (100%)	8 (80%)
**Aminosides**	Gentamycine (100%)	20	13 (86.66%)	---
	Kanamycine (100%)	20	13 (86.66%)	---
**Quinolones**	Ciprofloxacine	--	13 (86.66%)	6 (60%)
**Autres**	Lincomycine (100%)	20	13 (86.66%)	10 (100%)
	Erythromycine (100%)	20	13 (86.66%)	10 (100%)

N= nombre

## Discussion

Cette étude de 100 cas de sinusites maxillaires chroniques suppurées d'origine nasale permet quelques commentaires relatifs aux modalités de prélèvement, à l’épidémiologie bactérienne et à la sensibilité de ces germes aux antibiotiques.

**Les modalités de prélèvement:** Le prélèvement par aspiration à l'aide d'une seringue stérile à travers le trocart est la méthode recommandée car elle permet de rechercher les germes anaérobies et d’éviter les souillures par des germes de la fosse nasale.

**Epidémiologie bactérienne:** La fréquence des germes responsables des sinusites maxillaires chroniques varie d'un pays à l'autre. L'infection est souvent polymicrobienne [1]. Les germes anaérobies sont souvent rencontrés lorsque le prélèvement du pus a été obtenu par ponction du sinus et l'utilisation d'une bonne technique de recherche des anaérobies. Selon les études la proportion des germes anaérobie varie de 25 à 56% des germes [2–5].

Les germes anaérobies n'ont pas été systématiquement recherchés dans cette étude. Les cultures stériles ont été retrouvées dans 32% dans cette série, dans 30% dans la série de Pontal et al [6], et dans 14.28% dans la série de Sener et al [7]. Les cultures stériles peuvent s'expliquer par le non-respect des techniques de prélèvement, des délais d'acheminement et de traitement des échantillons, des difficultés liées à la recherche de certains germes et les antibiothérapies antérieures effectuées par certains patients. Le streptococcus pneumoniae (Pneumocoque) suivi du staphylococcus aureus étaient les germes les plus fréquents dans cette étude et dans celles de Kamau et al [8] au Kenya et de Kalcioglu et al en turquie [9], par contre le staphylococcus aureus était plus fréquents dans les séries de Gehanno [10], de Aneke [11] et Fairbanks [12].

**Sensibilité des germes isolés aux antibiotiques:** L’étude de la sensibilité des germes isolés aux antibiotiques détermine le choix de l'antibiotique dans le traitement des sinusites maxillaires chroniques. Les pneumocoques isolés dans notre série étaient tous sensibles à l'oxacilline, aux céphalosporines, à l’érythromycine et à la lincomycine. De plus en plus de pneumocoques sont résistants à la pénicilline G comme constaté dans cette étude et celle de Kales et al [13], Slack et al [14] et Kim et al [15].

Les staphylocoques dorés sont producteurs de bêta-lactamase ce qui explique leur résistance face à la pénicilline G [5, 15]. Les staphylocoques dorés sont souvent méticillino-sensible [15] et ont une bonne sensibilité aux aminosides, aux macrolides et aux quinolones [15]. Les souches de streptocoques du groupe A ont une bonne sensibilité à la pénicilline G [13], aux quinolones et aux macrolides [13]. Les souches de pseudomonas sont connues pour être naturellement résistantes à la plupart des antibiotiques [16] comme noté dans cette série. Les souches d'entérobactéries isolées ont été naturellement résistantes aux betalactamines en dehors des céphalosporines de 3^e^ génération.

## Conclusion

Les sinusites maxillaires chroniques suppurées constituent une affection fréquente chez l'adulte. Les germes responsables isolés après aspiration endosinusienne lors de la ponction du sinus étaient dominés par *Streptocoque pneumoniae*, *Staphylococcus aureus* et le streptocoque du groupe A. Les germes isolés avaient une bonne sensibilité aux quinolones et aux aminosides. L'utilisation des quinolones et des aminosides est préconisée en première intention devant une sinusite maxillaire chronique suppurée de l'adulte dans notre contexte.
